# Estimating completeness of national and subnational death reporting in Brazil: application of record linkage methods

**DOI:** 10.1186/s12963-020-00223-2

**Published:** 2020-09-04

**Authors:** Luiz Fernando Lima Costa, Marli de Mesquita Silva Montenegro, Dacio de Lyra Rabello Neto, Antonio Tadeu Ribeiro de Oliveira, Jose Eduardo de Oliveira Trindade, Tim Adair, Maria de Fatima Marinho

**Affiliations:** 1 Brazilian Institute of Geography and Statistics (IBGE), Level 8, 500 Republic of Chile Avenue, Rio de Janeiro, RJ 20031-170 Brazil; 2 Ministry of Health, SRTVN 701, Via W5 Norte, PO700 Building, 6th floor-DASNT, Brasilia, DF 70723-040 Brazil; 3 University of Melbourne, Melbourne School of Population and Global Health, The University of Melbourne, Level 5, Building 379, 207 Bouverie Street, Carlton, Victoria 3010 Australia; 4 Tele-Health/Federal University of Minas Gerais, Pres. Antônio Carlos, 6627-Pampulha, Belo Horizonte, MG 31270-901 Brazil

**Keywords:** Mortality, Registration completeness, Record linkage, Capture-recapture methods, Generalized linear modeling, Civil registration and vital statistics, Brazil

## Abstract

**Background:**

In Brazil, both the Civil Registry (CR) and Ministry of Health (MoH) Mortality Information System (SIM) are sources of routine mortality data, but neither is 100% complete. Deaths from these two sources can be linked to facilitate estimation of completeness of mortality reporting and measurement of adjusted mortality indicators using generalized linear modeling (GLM).

**Methods:**

The 2015 and 2016 CR and SIM data were linked using deterministic methods. GLM with covariates of the deceased’s sex, age, state of residence, cause of death and place of death, and municipality-level education decile and population density decile, was used to estimate total deaths and completeness nationally, subnationally and by population sub-group, and to identify the characteristics of unreported deaths. The empirical completeness method and Global Burden of Disease (GBD) 2017 estimates were comparators at the national and state level.

**Results:**

Completeness was 98% for SIM and 95% for CR. The vast majority of deaths in Brazil were captured by either system and 94% were reported by both sources. For each source, completeness was lowest in the north. SIM completeness was consistently high across all sub-groups while CR completeness was lowest for deaths at younger ages, outside facilities, and in the lowest deciles of municipality education and population density. There was no clear municipality-level relationship in SIM and CR completeness, suggesting minimal dependence between sources. The empirical completeness method model 1 and GBD completeness estimates were each, on average, less than three percentage points different from GLM estimates at the state level. Life expectancy was lowest in the northeast and 7.5 years higher in females than males.

**Conclusions:**

GLM using socio-economic and demographic covariates is a valuable tool to accurately estimate completeness from linked data sources. Close scrutiny of the quality of variables used to link deaths, targeted identification of unreported deaths in poorer, northern states, and closer coordination of the two systems will help Brazil achieve 100% death reporting completeness. The results also confirm the validity of the empirical completeness method.

## Introduction

Routine, accurate, and timely data on deaths are an essential source of evidence for national and subnational governments to monitor population health and develop programs to reduce disease and injury burden [1]. In Brazil, the fifth most populous country in the world, such information is especially important given its significant socio-economic and population health inequalities [2–4]. Such evidence is best served by a complete civil registration and vital statistics (CRVS) system [1].

However, there is no one reporting system that captures all deaths in Brazil. Two parallel death reporting systems exist: (1) the Civil Registry (CR), created by Law in 1874 under the control of the National Justice Council at the national level and justice courts at state level, for which the Brazilian Institute of Geography and Statistics (IBGE) has had responsibility for collating statistics since 1973, and (2) the Mortality Information System (SIM), created in 1975 and which is the responsibility of Ministry of Health (MoH). The two systems have different objectives: to provide inputs for the calculation of vital and epidemiological statistics (in the case of SIM), and, as required by the Public Records Act, the production of official information for the legal rights of each dying individual (in the case of the CR). Both sources commonly capture the majority of deaths in Brazil, but some events are recorded exclusively by only one of these sources [5].

The process for the recording of deaths differs by the place of occurrence and the availability of health professionals. For each death, the death declaration (DO) should be issued, which can only be signed by doctors. In rare places where there are no doctors, the presence of two witnesses and one civil registrar is required. The DO is a document prepared and developed by the MoH, which contains the International Form of Medical Certificate of Cause of Death, as well as variables of identification, socio demographic, such as age, gender, race, occupation, among others. The DO collects mortality information for statistical purposes and is also a legal document required by CR offices, as a Medical Certificate of Cause of Death, to comply with the Public Records Act. For deaths that occur in health facilities (72% of SIM deaths), facility staff report the deaths to the SIM system and provide a DO copy for the family to take to the CR office and obtain a death certificate to enable burial and take all legal measures regarding the death [6]. For home deaths that are attended by a health professional, the death is reported to local health authorities (usually the Municipality Health Department) and a DO is issued by a doctor. Thereafter, the process is the same as for facility deaths. If a health professional does not attend a home death nor know of the death (especially in more remote areas), the family may report it to the CR office with two witnesses and receive a death certificate. For each type of death, the death will not be registered if the family does not provide the DO nor report the death to the CR office.

Given that the CR and SIM operate parallel data collection systems, since 2006 the two sources have been able to be linked by a DO number and other variables. This record linkage has enabled application of direct or capture-recapture techniques to estimate the completeness of reporting of CR and of SIM; that is, the percentage of all actual deaths in Brazil reported by each source. An analysis of the linked dataset using generalized linear modeling (GLM) estimated that, for 2015, completeness of SIM reporting was 97% and completeness of CR reporting was 94% [7, 8].

Other estimates of the completeness of SIM have used the demographic technique death distribution methods, which measures completeness by assessing the consistency of the age pattern of the population and reported deaths and making specific assumptions about the dynamics of the population [9, 10]. One such study found completeness of SIM for the period 2000–2010 to exceed 90% for just over half of states, being lowest in northern states [11]. However, death distribution methods have a number of limitations, particularly that they are not very timely because they are most reliable when they estimate completeness of deaths occurring between two censuses, and also that they assume that populations only have limited migration, which means that their estimates for subnational areas are subject to considerable uncertainty [12, 13].

The linked CR and SIM datasets contain extensive information on the characteristics of the deceased and the death, which can be linked with census data of municipality-level characteristics. This study aims to use this linked data to measure completeness of SIM and CR reporting at the national and subnational level in Brazil. The above description of the SIM and CR systems implies that there is some dependence between the two sources. A considerable advantage of having a linked dataset is that GLM can be used with individual and area-level covariates to estimate death reporting completeness by minimizing bias from dependence between the two sources. This study also aims to use the GLM results to show evidence of which population sub-groups are least likely to have their deaths reported and so enable targeting of efforts to help find unreported deaths and improve the coverage and operation of these systems across Brazil. The GLM estimates of completeness are also compared with other methods to estimate completeness: the Chandrasekar-Deming method, the recently developed empirical completeness method and completeness estimated from the Global Burden of Disease study [12, 14, 15]. The study also uses the GLM results to estimate national and subnational mortality indciators.

## Methods

### Data sources and record linkage

The study assessed deaths occurring in 2015 and 2016 and reported by the CR and SIM [6, 16, 17]. Each source contained variables with information about the death and the deceased: sex, age, place of death (i.e., hospital, household, public), if the cause of death was unnatural (i.e., injuries) or natural (i.e., all other deaths), and the deceased’s municipality of residence. Municipality-level variables of the percentage of the population aged 25–39 years that had completed high school and population density were calculated using the 2010 Census 1[18]. The CR and SIM data were linked by deterministic methods [5]. In 2016, 94% of reported deaths were found in both databases, with 2% in the CR only and 4% in the SIM only. More details of the record linkage are shown in Additional file 1 (with results in Supplementary Table 1, Additional file 1).

### Capture-recapture methods

Capture-recapture methods estimate completeness of each of two linked data sources. The most commonly used capture-recapture method is that developed by Chandrasekar and Deming (C-D). The C-D method uses two linked data sources to estimate the total number of events in a population, assuming the two sources are independent (that is, likelihood of a death being captured in one source is influenced by the likelihood of it being captured in the other source), there is equal probability of all deaths being captured in each data source, the population is closed, and there is accurate reporting of information about the death and accurate linkage of the databases [14]. Further details of the C-D method are described in Additional file 2.

GLM estimates completeness of linked datasets using a regression approach. The GLM method, developed by Huggins, models the probability of capture by either source according to observable covariates [19, 20]. GLM has prevously been used to estimate birth registration completeness in Brazil, as well as to estimate completeness of the linked SIM and CR data in Brazil but without including any covariates of characteristics of the death or deceased [7, 19–21].

This study used the GLM method. Consider the following model equation: [20, 21]

$$ \mathit{\ln}\left(\frac{p_{ib}}{1-{p}_{ib}}\right)={\beta}_0+\sum \limits_{j=1}^k{\beta}_j{x}_j $$

where

*p*_*ib*_ is the probability of individual *i* being in each database *b* (CR and SIM)

*β*_0_ is the intercept

*β*_*j*_ is the parameter for the *j*th variable, *j* = 1,2,..,*k*

*x*_*j*_ is the *j*th variable, *j* = 1,2,..,*k*

*i* = 1,2,3,…,*N* which is the number of death records

*b* = 1,2 which is the number of sources

*k* is the number of covariates in the model

To estimate the number of deaths, we use the estimated probability of being captured calculated by the model above using the following steps:

$$ 1\Big)\ \alpha ={\hat{\beta}}_0+\sum \limits_{j=1}^k{\hat{\beta}}_j{x}_j $$

$$ 2\Big)\ {p}_{ib}^{\ast }=\frac{e^{\alpha }}{e^{\alpha }+1} $$

$$ 3\Big)\ {\hat{p}}_i=1-\prod \limits_{i=1}^N\left(1-{p}_{ib}^{\ast}\right) $$

$$ 4\Big)\ \hat{N}=\sum \limits_{i=1}^N\frac{1}{{\hat{p}}_i} $$

where

${\hat{\beta}}_0$ and ${\hat{\beta}}_j$are the parameter estimates resulted from the model above

${p}_{ib}^{\ast }$ is the estimated probability of individual *i* being captured in source *b*

${\hat{p}}_i$ is the probability of individual *i* being captured at least once by either source

$\hat{N}$ is the estimated number of deaths. For each individual death, it is the estimated number of deaths per reported death—if there is a low probability of a reported death being captured in either source, then there will be a higher number of estimated deaths per that individual reported death. From this figure, the completeness of each source can be calculated. The Akaike Information Criteria (AIC) was used to choose the most appropriate model according to the covariates included, while multicollinearity of the covariates was also assessed [22].

GLM minimizes the bias of the C-D method when the linked data sources are dependent and have heterogeneous capture probabilities. It does this by estimating the number of deaths (and therefore completeness) for each combination of covariates in the model; that is, deaths with homogenous characteristics (e.g., age, sex, place of death) and for which dependency and heterogeneity of capture probabilities between SIM and CR would be lower than for all deaths in the population. This is equivalent to Chandrasekar and Deming’s recommendation to estimate total events separately for homogenous groups to reduce dependency bias [14]. By estimating completeness for different combinations of variables, we can also better understand the operation of each system and identify which population sub-groups account for the highest percentage of deaths missed by both systems, to inform further efforts to capture these deaths.

The estimated completeness of each source according to the GLM method is presented at the national and state level, as well as for other selected characteristics. The national estimate of completeness of each source is also compared to the C-D method. The number of deaths unreported by either source is estimated according to different covariates; such information can help target efforts to attain 100% completeness. To assess if the sources are independent, we compare the completeness estimates for CR and SIM for the 5570 municipalities in Brazil level using a scatterplot and measuring the *r*^2^ of their relationship; close correlation between completeness estimates would indicate source dependence. Key mortality indicators (life expectancy at birth and adult mortality per 1000 or *45q15*) based on total estimated deaths and population estimates are presented nationally and for each state [23].

### Empirical completeness method

The empirical completeness method estimates completeness of death reporting using data inputs that capture the expected relationships among the principal determinants of mortality levels in a population [12]. The method was developed based on 2451 country-years in 110 countries between 1970 and 2015 in the GBD database. The method predicts the logit of completeness with data inputs of the registered crude death rate (registered deaths per 1000 population), registered crude death rate squared, the natural log of under-five mortality (*5q0*), the percentage of the populations aged 65 years and above, completeness of under-five death registration and country-level random effects. Two versions of the method have been developed: model 1 which includes all these variables, and model 2 which excludes under-five completeness for populations where this is not representative of completeness across all ages. The method overcomes the limitations of existing completeness methods (especially death distribution methods), including reliance on often unrealistic assumptions about population dynamics and lack of timeliness. The accuracy of the *5q0*, particularly at the subnational level, is important; we use the GBD’s estimate of state- and national-level *5q0* for Brazil [15].

### Global Burden of Disease

The GBD estimates total deaths for Brazil and each of its states [15]. The GBD methods are described in detail elsewhere; mortality rates and hence estimated total deaths are estimated by a model life table that uses *5q0* and *45q15* estimates as inputs [24]. Time trends in under-five mortality are modeled using estimates from various surveys (e.g., National Household Sample Health Survey, National Demographic and Health Survey of Children and Women), censuses (e.g., 2000, 2010), and registered under-five deaths, while time trends in adult mortality are modeled primarily using completeness-adjusted registered deaths. The GBD and empirical method models 1 and 2 are compared with GLM estimates using the root mean squared difference.

## Results

**Fig. 1:**
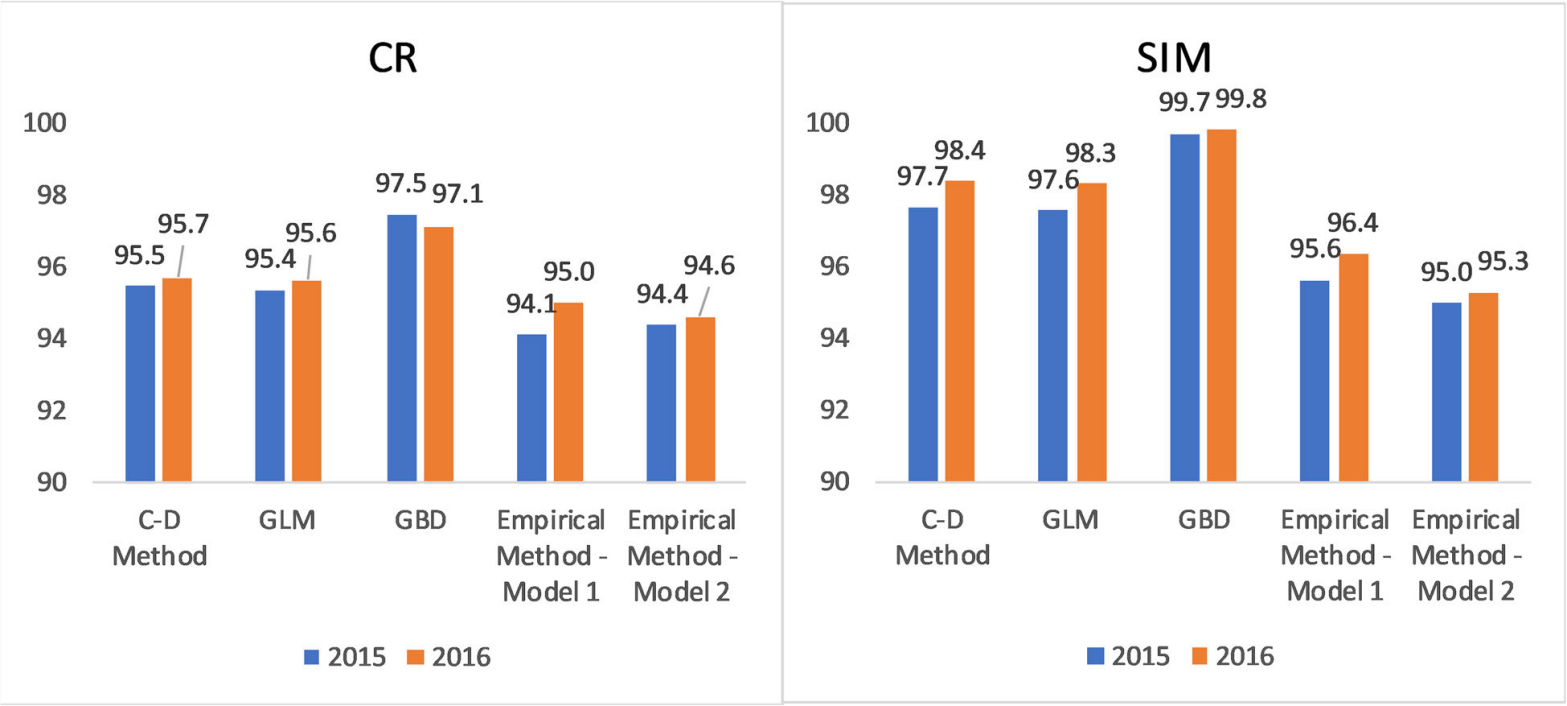
Completeness of death reporting (%) by source and method, Brazil, 2015 and 2016

Completeness of death reporting in Brazil is estimated to be very high for each system. According to the GLM, in 2016 completeness of SIM data was 98% and CR data was 96% (Fig. 1). The GBD’s completeness estimates were highest (almost 100% for SIM) and the empirical method’s estimates were lowest. An increase in completeness from 2015 to 2016 was observed for each system, which demonstrates the improvement of data collection. The concordance of the estimates across the methods results in a quite narrow range of estimates of total deaths in Brazil (in 2016 ranging from 1,312,303 for the GBD method to 1,374,660 for the empirical method model 2 applied to the SIM data) (Supplementary Table 2, Additional file 3).

Notably, the GLM estimates only 1929 deaths in Brazil in 2016 were not reported by either system or only 0.1% of estimated total deaths according to this method. A high 94% of all deaths in the country were captured by both sources (1,253,284/1,332,398) according to the GLM. The model results are shown in Supplementary Tables 3 and 4, Additional file 3. The strongest model according to the AIC was that which included all the available variables. No multicollinearity was found in this strongest model.

**Fig. 2:**
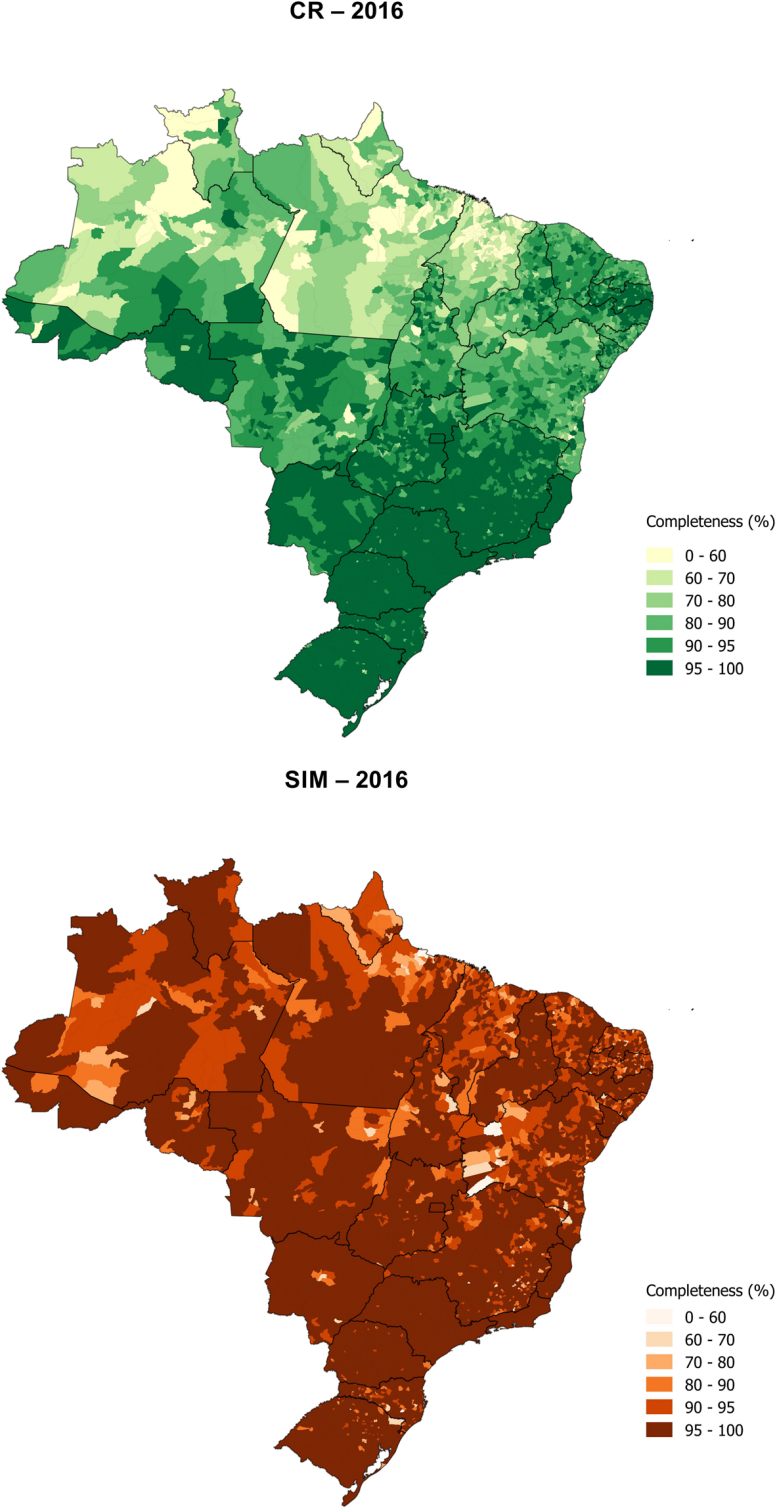
Estimate of completeness using GLM by municipality, SIM and CR, Brazil, 2016

The GLM estimates show that completeness of SIM death reporting was high in each state, but CR completeness is relatively low in specific states. In 2016, the same four states had the lowest completeness of CR and SIM death reporting: Maranhão (72% CR, 94% SIM), Amapá (76% CR, 93% SIM), Pará (81% CR, 96% SIM), and Roraima (87% CR, 95% SIM). Completeness reached 99.5% for CR in Sao Paulo and Distrito Federal and 99.9% for SIM in Sao Paulo. All states’ completeness can be seen in Table 1 (2016) and Supplementary Table 5, Additional file 3 (2015). Municipality-level completeness showed that, for each system, completeness was lowest in the north of Brazil (Fig. 2).

**Table 1:** Completeness of death reporting (%) by source and method, Brazil and each state, 2016

State	CR				SIM			
	GLM	Empirical model 1	Empirical model 2	GBD	GLM	Empirical model 1	Empirical model 2	GBD
Brazil	95.6	95.0	94.6	97.1	98.3	96.4	95.3	99.8
Rondônia	98.4	97.3	94.9	96.7	97.7	97.4	95.0	96.1
Acre	95.4	90.2	87.5	98.9	97.5	90.2	88.3	101.1
Amazonas	89.1	92.1	90.0	91.6	96.9	96.1	92.0	99.6
Roraima	86.7	89.5	86.0	88.0	95.0	94.4	88.8	96.5
Pará	81.3	88.1	88.0	82.8	95.6	96.2	92.5	97.3
Amapá	75.9	82.8	83.6	81.2	92.6	95.0	88.9	99.0
Tocantins	90.8	90.5	89.5	89.4	97.0	92.9	91.4	95.6
Maranhão	71.5	72.7	78.8	70.6	93.7	90.7	88.8	92.6
Piauí	87.8	84.4	88.4	87.9	98.3	94.2	92.0	98.3
Ceará	92.6	90.6	91.8	94.7	96.4	93.3	93.0	98.6
Rio Grande do Norte	89.1	94.9	94.8	94.8	95.8	97.5	96.0	101.9
Paraíba	96.0	96.7	96.2	100.6	96.2	97.3	96.5	100.9
Pernambuco	95.8	94.7	95.3	96.9	98.4	96.6	96.2	99.5
Alagoas	91.6	95.7	95.5	95.2	95.8	97.4	96.2	99.6
Sergipe	92.1	92.4	93.0	92.7	98.5	95.8	94.6	99.2
Bahia	91.6	91.4	91.2	89.9	95.8	94.6	92.5	94.1
Minas Gerais	98.3	95.3	94.5	99.9	98.5	95.8	94.9	100.2
Espírito Santo	99.3	95.6	94.4	101.4	98.9	96.0	94.6	101.1
Rio de Janeiro	98.3	98.2	97.2	100.5	99.5	98.3	97.5	101.7
São Paulo	99.5	97.3	96.3	100.8	99.9	97.4	96.5	101.3
Paraná	98.8	96.7	95.8	99.9	99.5	96.9	96.1	100.5
Santa Catarina	97.5	90.8	92.6	99.6	98.4	91.7	93.2	100.5
Rio Grande do Sul	99.4	98.5	98.3	101.3	99.3	98.5	98.4	101.2
Mato Grosso do Sul	97.3	98.1	94.2	101.5	98.3	98.4	94.7	102.6
Mato Grosso	90.5	95.5	92.4	93.3	98.0	97.8	94.2	101.0
Goiás	96.2	95.4	94.9	97.1	98.8	96.4	95.5	99.7
Distrito Federal	99.5	93.2	90.0	99.2	99.4	93.2	90.4	99.1
Root mean squared difference with GLM	-	2.8	3.2	2.1	-	2.4	3.8	2.3

Nationally, for each source, the empirical method model 1 and GBD completeness estimates were each within two percentage points of the GLM estimates (Table 1). Across the states, the root mean squared difference (RMSD) of CR completeness for empirical method model 1 and GBD compared with the GLM estimates was low for each method in 2016, with the GBD being slightly lower (2.1 percentage points for GBD, 2.8 for empirical method model 1, 3.2 for empirical method model 2). However, in 2015, the empirical method model 1 RMSD was slightly lower for SIM completeness while the GBD RMSD was marginally lower for CR completeness (Supplementary Table 5, Additional file 3). A notable finding was that GBD estimated completeness of SIM reporting exceeded 100% for 12 states in 2016.

**Fig. 3:**
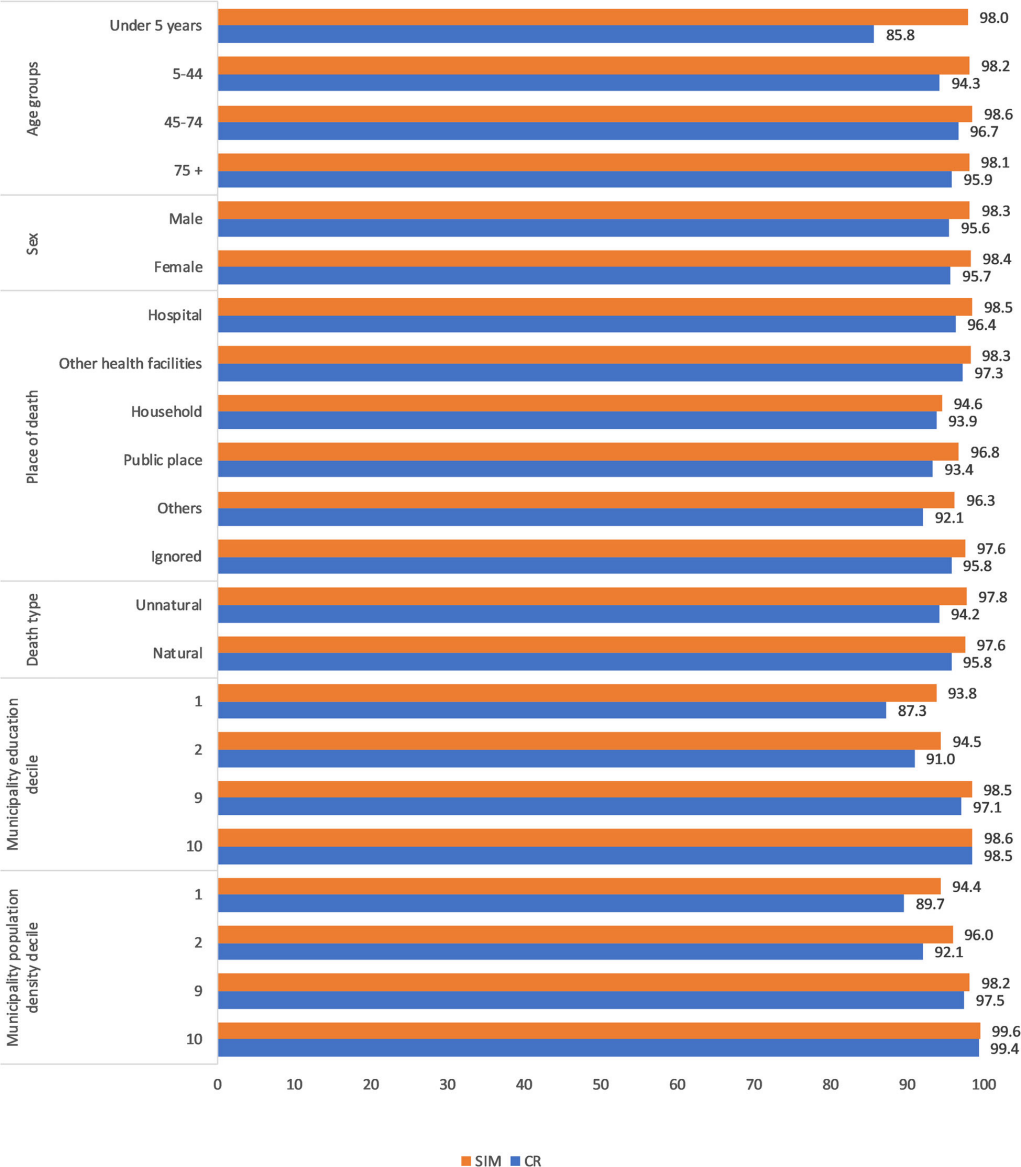
Completeness of death reporting (%) by source using GLM, by selected population sub-groups, 2016

The GLM allows estimation of completeness for other population sub-groups (Fig. 3). The completeness of SIM death reporting was consistently high across all sub-groups, varying little by sex, cause of death and age. It was lowest for deaths in households, municipalities in the lowest two education deciles, and municipalities in the lowest population density decile, where it still was above 93% in 2016. In contrast, completeness of CR death reporting was particularly low for deaths at younger ages, being less than 90% at ages less than 5 years, deaths outside facilities, and the lowest deciles of municipality education and population density. Completeness estimates were made for every population sub-group included in the GLM (Supplementary Table 6, Additional file 3).

**Fig. 4:**
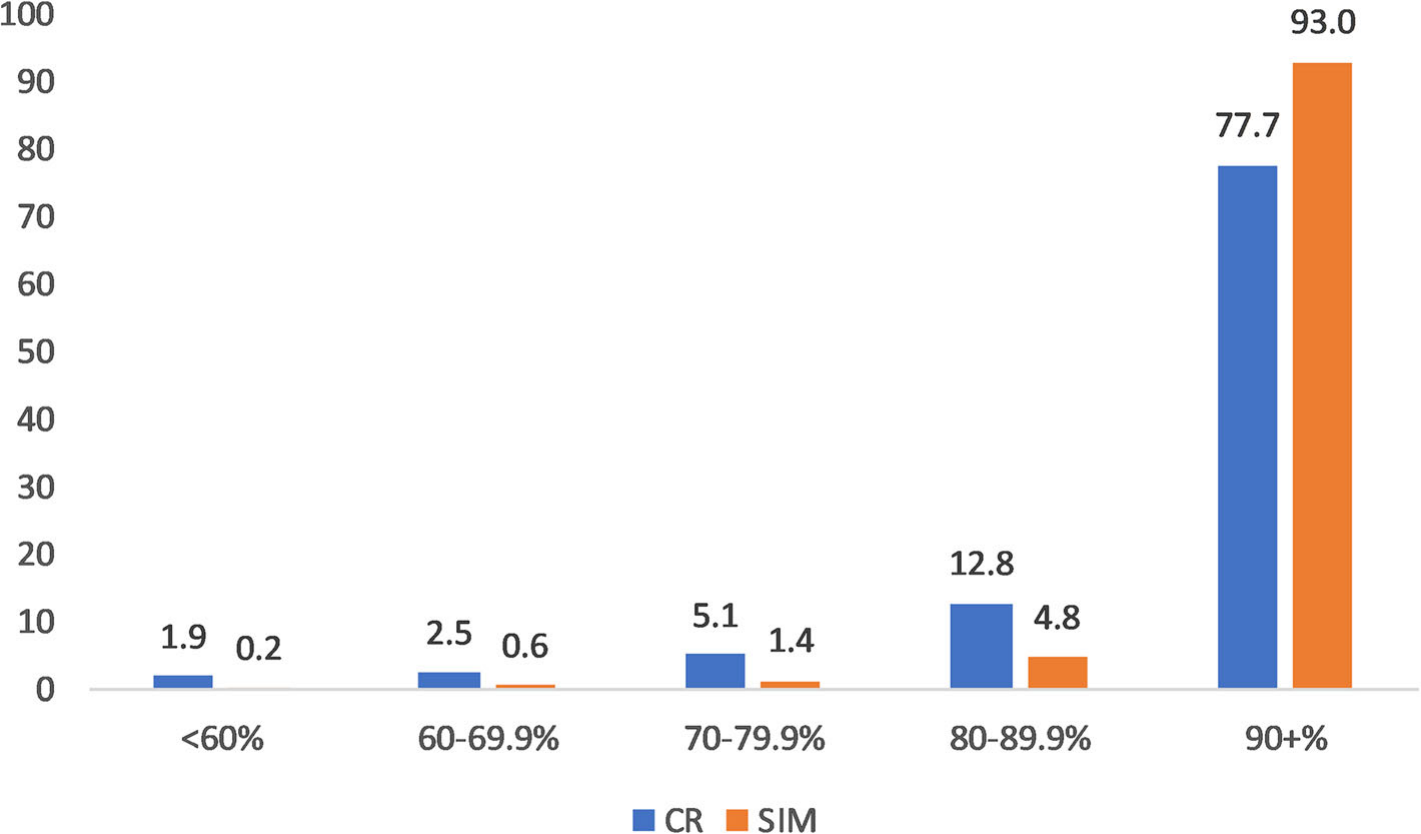
Percentage distribution of municipalities by completeness of death reporting (%) and source using GLM, 2016 here

The consistently high completeness of SIM death reporting is demonstrated by 93% of municipalities with completeness of SIM death reporting of over 90%, compared with 78% of municipalities for completeness of CR death reporting (Fig. 4). SIM completeness was at least 99% in 48% of municipalities and CR completeness at least 99% in 36% of municipalities. Completeness of less than 80% was rare for each data source, particularly for SIM where it was found in only 2% of municipalities compared with 10% for CR.

A valuable application of the GLM method is to estimate the characteristics of the deaths not reported by either system. Of those deaths estimated to be unreported, about two-thirds were in the states of Pará, Maranhão, and Bahia, about half were in municipalities in the two lowest population density deciles and about two-thirds were in municipalities in the two lowest education deciles (Table 2). The percentage of missing and reported deaths that are in each category are presented (Supplementary Table 7, Additional file 3).

**Table 2:** Estimated deaths not captured using GLM, by selected population sub-groups, Brazil, 2015 and 2016

Category	2015		2016	
	Estimated deaths	%	Estimated deaths	%
Total deaths not captured by either system	2705	100	1929	100
State—Pará	413	15.3	304	15.8
State—Maranhão	694	25.7	530	27.5
State—Bahia	385	14.2	304	15.9
Age—85+	606	22.4	445	23.1
Place of death—hospital	896	33.1	622	32.2
Place of death–household	1336	49.4	984	51.0
Cause of death—natural death	2384	88.1	1702	88.2
Municipality—1st decile of population density	870	32.2	644	33.4
Municipality—2nd decile of population density	573	21.2	411	21.3
Municipality—1st decile of education level	1032	38.2	767	39.8
Municipality—2nd decile of education level	746	27.6	518	26.9

**Fig. 5:**
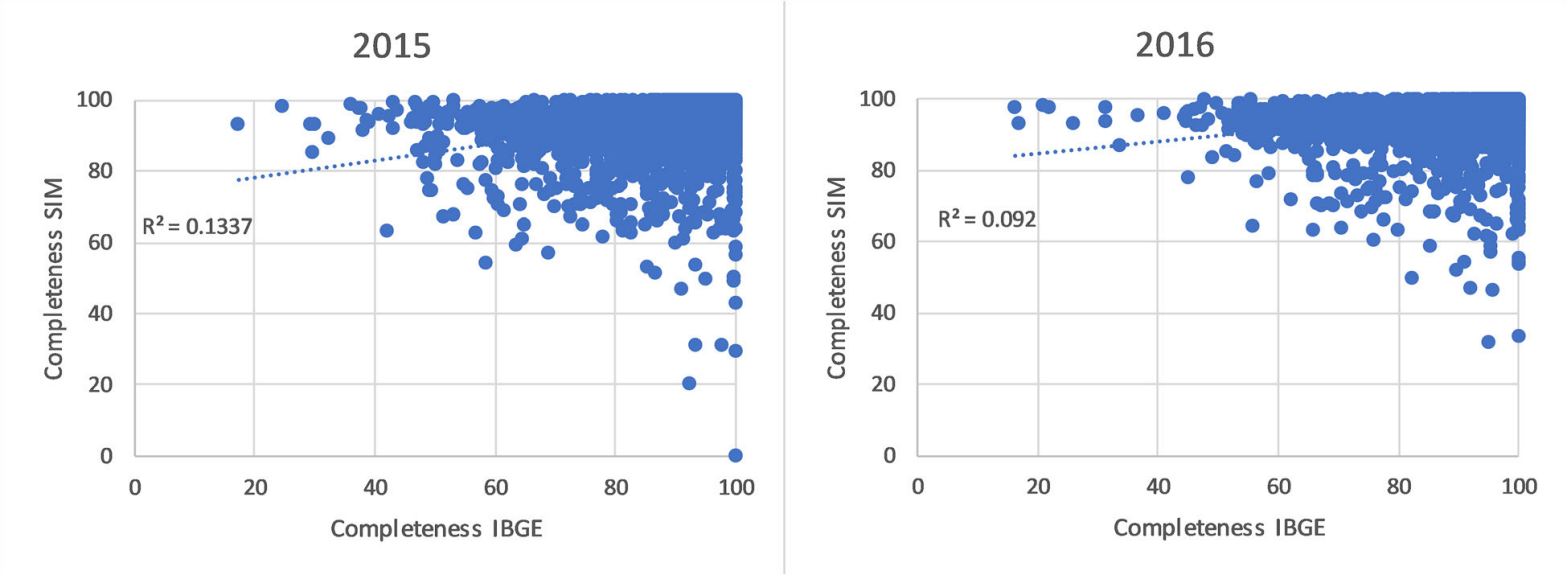
Municipality completeness of death reporting (%) using GLM, CR by SIM, Brazil, 2015 and 2016

As mentioned, the design of the two systems suggest they may have some dependence. However, evidence from the GLM suggests this is not the case. The GLM estimates of completeness were only within 0.1% of the C-D estimates (which assumes complete independence), therefore the latter is not significantly biased and hence suggests very little dependence between the CR and SIM databases. Figure 5 shows completeness of the two sources does not show a clear relationship in either year; the *r*^2^ was only 0.13 in 2015 and 0.09 in 2016. If there was strong dependence between sources—i.e., the likelihood of a death being in one source was influenced by the likelihood of it being in another source—then a much closer municipality-level relationship in completeness would be expected.

The deaths estimated by the GLM method were used to calculate key mortality indicators (Table 3). Life expectancy of females (79.4 years) was 7.5 years higher than for males (71.9), while female adult mortality (91) was less than half that of males (190). Life expectancy at birth was lowest and male adult mortality highest in the northeast states of Alagoas, Pernambuco, and Sergipe, while female adult mortality was also high in Rio de Janeiro (Supplementary Table 8, Additional file 3).

**Table 3:** Mortality indicators calculated using GLM, by sex, Brazil, 2016

Mortality indicators	Both	Males	Females
Life expectancy at birth	75.7	71.9	79.4
Adult mortality rate (45q15), per 1000	141	190	91

## Discussion

Application of GLM to linked SIM and CR death records in Brazil confirm a high and increasing level of death reporting completeness for each system. The completeness of the SIM reporting system in 2016 was 98% and CR was 96%; combined, the two sources account for all but 2000 of the estimated 1.33 million annual deaths in Brazil. Although there is some dependence between the systems, as demonstrated by their design and that 94% of estimated deaths are captured by both systems, the similarity in estimated total deaths between the C-D method and GLM, as well as the lack of correlation between municipal estimates of completeness of CR and SIM, shows that any bias from dependency is minimal. The estimated deaths according to GLM shows that, in 2016, female life expectancy (79.4 years) was substantially higher than for males (71.9 years), and male adult mortality (190 per 1000) was double that of females (91 per 1000).

The GLM results concord quite closely with those of the empirical method and the GBD. State-level completeness estimates of the empirical method and GBD were each within 2–3 percentage points of the GLM, although the GBD estimates exceeded 100% for some states. For the empirical completeness method, which relies on relatively few data inputs, the results confirm its validity and demonstrate that it can be used with confidence across a range of settings. The state-based estimates provided are an improvement on past estimates that have relied on death distribution methods which, among other limitations, assume little migration between subnational areas and only measure completeness for intercensal periods, which the most recent for Brazil is 2000 to 2010.

The strength of the SIM reporting system is that it has high completeness across almost all population sub-groups, whether by age group, state, or municipality-level education or population density. The only relatively low completeness for SIM (i.e., less than 95%) is found for deaths at home and in lower education and population density municipalities, which is to be expected given these deaths are least likely to be attended by a doctor. The CR reporting system is least likely to register deaths of infants and children, those occurring outside facilities, and in occurring the lowest education and more remote municipalities. Low reporting of younger deaths may be because the births were not registered or because there is no imperative to register these deaths for the purposes of inheritance or life insurance. The poorer completeness in more remote municipalities may reflect the greater distance from registration offices in those locations and possibly lower awareness of the requirement to register deaths. The states where CR is estimated to have the lowest completeness—Maranhao, Piaui, Amapa—are located in the north of the country. In other states comprising more remote riverside populations, such as Amazonas, completeness is not substantially lower than the national level; Fig. 2 does show that some municipalities in Amazonas have low completeness, but such remote populations are a relatively small proportion of the state’s total population.

The findings are particularly useful in that they allow targeting of the missing deaths within population sub-groups, with the majority occurring in the states of Pará, Maranhão, and Bahia, and municipalities with the lowest education levels and those that have low population density. Such a targeted approach can help Brazil attain 100% completeness. In the past, proactive search of deaths from various sources such as community health agents, registry offices, official and non-official cemeteries, and funeral homes has been conducted in municipalities where completeness is particularly low; a 2008 study found that 28% of non-reported deaths were found in sources such as non-official cemeteries [25].

The GLM approach has many advantages over other methods to estimate completeness, such as providing a reliable estimate of completeness because it controls for factors that contribute to dependency between sources, a potential issue when estimating completeness using capture-recapture methods. It also allows for estimation of completeness and the number of missing deaths according to the categories of the covariates in the model. This information is vital to understanding the operation of the system according to socio-economic, geographic, and system (e.g., place and cause of death) factors, as well as to inform targeting of strategies to report missing deaths and to attain full completeness. Various Latin American countries that have multiple incomplete routine sources of mortality data, including Peru, Paraguay, and Honduras, could use the GLM approach to estimate completeness and total deaths. For example, in Peru, death reporting from Ministry of Health and the National Registry of Identification and Civil Status (RENIEC), as well as other mortality sources (e.g., army hospitals), could be linked to facilitate an analysis similar to that presented here. This would be an valuable interim measure while the National Death Information System (SINADEF), which is integrating Ministry of Health and RENIEC data, only covers a portion of the county.

A potential limitation of the GLM used in this study is that data of other factors that are expected to predict completeness and contribute to source dependency were not included, such as individual socio-economic factors (e.g., education and income) and more detailed causes of death, and so completeness estimates could be biased. However, although the design of the two systems does suggest a degree of dependency, the significant lack of correlation in municipal estimates of completeness of SIM and CR is such that more granular information about decedents, much of which is already measured at the municipality level, is unlikely to reduce bias more than a small amount. Another limitation is that the estimated completeness for each category of the reported covariates does not provide direct information on why these deaths are not being reported (e.g., are families not reporting the death to civil registry office or is there “leakage” of deaths during reporting from the local to national level).

Furthermore, additional examination of the results revealed that, in some of the more remote and lower socio-economic municipalities, where a high proportion of missing deaths occur, estimated total deaths from the GLM was significantly lower (and hence completeness is higher) than that estimated by the C-D method. This could be due to the education decile of the municipality being an inferior measure of socio-economic status than individual or household education or income, which means that there are potentially more missing deaths than estimated. However, it could be that in such municipalities, there are data quality issues, especially related to DO number, age, or place of residence, which result in less deaths being linked than there should be and so the C-D method underestimates completeness. In these municipalities, the data quality of records to link the two systems should be closely investigated to identify if a less restrictive linkage criteria could be used and to improve estimates of the number of unreported deaths.

## Conclusions

This study has demonstrated the significant utility of applying a GLM approach to estimate completeness of death records linked from two systems to provide evidence of the performance of each system and to identify the number of unreported deaths. Both IBGE and MoH are aiming to achieve 100% completeness, and both institutions are united in discussing how best to advance this goal through harmonization of the two systems. The methods used here can be applied to birth statistics, extending previous work [21]. An important use is the estimation of statistical correction factors generated by each of the institutions (MoH and IBGE), in order to present convergent values of the indicators calculated by both institutions. More generally, the GLM approach can be employed where there are two linked reporting systems, especially where there are a number of covariates. The study also shows that the empirical completeness method, which relies on relatively limited data inputs and is less complex to implement than the GBD methods, is accurate compared when its results are with the GLM estimates and so provides confidence in its application across a wide range of settings.

## Supplementary information

**Additional file 1.** Record linkage. **Supplementary Table 1:** Results from linkage of SIM and CR death records, Brazil, 2015 and 2016.

**Additional file 2.** Chandrasekar-Deming method.

**Additional file 3: Additional Tables. Supplementary Table 2.** Reported deaths, estimates of total and unreported deaths and completeness of death reporting (%) by source and method, Brazil, 2015 and 2016. **Supplementary Table 3.** Results of GLM models of CR death reporting, 2015 and 2016. **Supplementary Table 4.** Results of GLM models of SIM death reporting, 2015 and 2016. **Supplementary Table 5.** Completeness of death reporting by source and method, Brazil and each state, 2015. **Supplementary Table 6.** Completeness of death reporting by source using GLM, by various population-sub-groups, 2015 and 2016. **Supplementary Table 7.** Estimated deaths not captured by either system, captured by CR and captured by SIM using GLM (%), by various population sub-groups, 2015 and 2016. **Supplementary Table 8.** Key mortality indicators calculated using GLM estimated deaths, by sex and state, Brazil, 2016.

**Additional file 4.** Number of deaths by source, Brazil and each state, 2015 and 2016.

## Data Availability

The number of deaths by residence state of deceased reported in CR, SIM, or both sources is shown in Additional file 4. More detailed data on linked individual deaths cannot be provided because of confidentiality policies of Ministry of Health, Brazil, and the Brazil Institute of Geography and Statistics.

## Abbreviations

C-D: Chandrasekar-Deming
CR: Civil Registry
CRVS: Civil registration and vital statistics
DO: Death declaration
GLM: Generalized linear modeling
MoH: Ministry of Health
IBGE: Institute of Geography and Statistics
SIM: Mortality Information System

## Footnotes

1 The SIM data also included characteristics of the individual, such as race, education level, and occupation, as well as the underlying cause of death according to the International Classification of Diseases Version 10 (ICD-10), but these were not available in the CR data and so could not be used. Where there were linked deaths with inconsistent data for the variables, the information reported in the SIM was used because it had less cases of “ignored” answers in each variable.
