# Supplementary material for: Estimating completeness of national and subnational death reporting in Brazil: application of record linkage methods
Source: Popul Health Metr. 2020 Sep 4;18:22. doi: 10.1186/s12963-020-00223-2 (PMC7650525; doi:10.1186/s12963-020-00223-2)
Supplement: Supplementary file 1 — Additional file 1. Record linkage. Supplementary Table 1: Results from linkage of SIM and CR death records, Brazil, 2015 and 2016. [file 12963_2020_223_MOESM1_ESM.docx]

**Additional File 1: Record linkage**

The linkage of SIM and CR data was conducted in two steps: firstly, using only the DO number and, secondly, with the remaining records, using a unique pairing key for each record. The pairing key was constructed using common variables in the two data sources: date of death, sex, age, state of residence of the deceased, municipality of residence of the deceased, place of death and part of the DO number. Only the records with an identical pairing key were considered a match. Supplementary Table 1 shows that a high proportion of deaths were matched between the two sources, with the SIM database having a higher number of unmatched deaths (and hence reporting more deaths overall) than the CR database.

**Supplementary Table 1: Results from linkage of SIM and CR death records, Brazil, 2015 and 2016**

|  | **2015** | | **2016** | |
| --- | --- | --- | --- | --- |
| **Database** | **Number** | **%** | **Number** | **%** |
| Both databases | 1,206,743 | 93.3 | 1,253,284 | 94.2 |
| CR only | 29,029 | 2.2 | 20,697 | 1.6 |
| SIM only | 57,432 | 4.4 | 56,488 | 4.3 |
