# Supplementary material for: Estimating completeness of national and subnational death reporting in Brazil: application of record linkage methods
Source: Popul Health Metr. 2020 Sep 4;18:22. doi: 10.1186/s12963-020-00223-2 (PMC7650525; doi:10.1186/s12963-020-00223-2)
Supplement: Supplementary file 2 — Additional file 2. Chandrasekar-Deming method. [file 12963_2020_223_MOESM2_ESM.docx]

**Additional File 2: Chandrasekar-Deming method**

The Chandrasekar-Deming method estimates the number of deaths not captured by either system using this equation:

$Y=\frac{N1*N2}{C}$

where *N1* is deaths reported in Source 1 but not Source 2, *N2* is deaths reported in Source 2 but not Source 1, *C* is deaths reported in both sources and *Y* is estimated deaths not reported by either source. The total estimated deaths in the population is simply the sum of *N1*, *N2*, *Y* and *C*. Completeness reporting is the number of deaths reported by a source divided by total estimated deaths. The accuracy of estimates of the C-D method is dependent on the following pre-requisites:

- Independence of data sources. Dependence between sources occurs when the likelihood of a death being captured in one source is influenced by the likelihood of it being captured in the other source; or deaths not reported by either source are not a random sample of all deaths.[1, 2] Complete independence between systems of parallel death reporting systems such as in Brazil is unlikely.
- Equal probability of all death being captured in each data source, which means that characteristics such as age, sex and geography do not exclude a death being captured by either source.[3]
- Closed population, i.e., no migration in the population between the death being captured by the two sources.
- Accurate reporting of information about the death and accurate linkage of the databases. The quality of the data linkage is very important, and there should be minimal missing data and accurate information about the records as sex, ages, place of residence, etc.

Once deaths have been linked, the C-D method is quite straightforward to apply. It has been used to estimate completeness of death registration or reporting in a number of settings, including Thailand, Vietnam, Oman, Kiribati and Bohol, Philippines.[3-8]

1. Chandrasekar C, Deming W: **On a method of estimating birth and death rates and the extent of registration.** *Journal of American Statistics Association* 1949, **44:**101-115.

2. Hook E RR: **Capture–recapture methods in epidemiology: methods and limitations.** *Epidemiologic Reviews* 1995, **17**.

3. Rao C, Kelly M: **Overview of the principles and international experiences in implementing record linkage mechanisms to assess completeness of death registration.** vol. Technical Paper No. 2017/5. New York: Population Division, Department of Economic and Social Affairs, United Nations; 2017.

4. Al Muzahmi SNK: **Mortality patterns in Oman: Demographic and epidemiological review.** *PhD Thesis.* The University of Queensland, School of Population Health; 2015.

5. Carter KL, Baiteke T, Teea T, Tabunga T, Itienang M, Rao C, Lopez AD, Taylor R: **Mortality and life expectancy in Kiribati based on analysis of reported deaths.** *Popul Health Metr* 2016, **14:**3.

6. Carter KL, Williams G, Tallo V, Sanvictores D, Madera H, Riley I: **Capture-recapture analysis of all-cause mortality data in Bohol, Philippines.** *Popul Health Metr* 2011, **9:**9.

7. Hoa NP, Rao C, Hoy DG, Hinh ND, Chuc NT, Ngo DA: **Mortality measures from sample-based surveillance: evidence of the epidemiological transition in Viet Nam.** *Bull World Health Organ* 2012, **90:**764-772.

8. Vapattanawong P, Prasartkul P: **Under-registration of deaths in Thailand in 2005-2006: results of cross-matching data from two sources.** *Bull World Health Organ* 2011, **89:**806-812.
