# Supplementary material for: Estimating completeness of national and subnational death reporting in Brazil: application of record linkage methods
Source: Popul Health Metr. 2020 Sep 4;18:22. doi: 10.1186/s12963-020-00223-2 (PMC7650525; doi:10.1186/s12963-020-00223-2)
Supplement: Supplementary file 3 — Additional file 3: Additional Tables. Supplementary Table 2. Reported deaths, estimates of total and unreported deaths and completeness of death reporting (%) by source and method, Brazil, 2015 and 2016. Supplementary Table 3. Results of GLM models of CR death reporting, 2015 and 2016. Supplementary Table 4. Results of GLM models of SIM death reporting, 2015 and 2016. Supplementary Table 5. Completeness of death reporting by source and method, Brazil and each state, 2015. Supplementary Table 6. Completeness of death reporting by source using GLM, by various population-sub-groups, 2015 and 2016. Supplementary Table 7. Estimated deaths not captured by either system, captured by CR and captured by SIM using GLM (%), by various population sub-groups, 2015 and 2016. Supplementary Table 8. Key mortality indicators calculated using GLM estimated deaths, by sex and state, Brazil, 2016. [file 12963_2020_223_MOESM3_ESM.docx]

**Additional File 3: Additional Tables**

**Supplementary Table 2: Reported deaths, estimates of total and unreported deaths and completeness of death reporting (%) by source and method, Brazil, 2015 and 2016**

|  | **2015** | **2016** |
| --- | --- | --- |
| ***Reported deaths*** |  |  |
| Both sources | 1,206,743 | 1,253,284 |
| CR only | 29,029 | 20,697 |
| SIM only | 57,432 | 56,488 |
| Any source | 1,293,204 | 1,330,469 |
| ***Estimated deaths and completeness*** |  |  |
| **C-D** |  |  |
| Total deaths | 1,294,586 | 1,331,402 |
| Deaths unreported by CR | 58,814 | 57,421 |
| Completeness – CR (%) | 95.5 | 95.7 |
| Deaths unreported by SIM | 30,411 | 21,630 |
| Completeness – SIM (%) | 97.7 | 98.4 |
| Deaths unreported by both sources | 1,382 | 933 |
| **GLM** |  |  |
| Total deaths | 1,295,909 | 1,332,398 |
| Deaths unreported by CR | 60,137 | 58,411 |
| Completeness – CR (%) | 95.4 | 95.6 |
| Deaths unreported by SIM | 31,734 | 22,620 |
| Completeness – SIM (%) | 97.6 | 98.3 |
| Deaths unreported by both sources | 2,705 | 1,923 |
| **GBD** |  |  |
| Total deaths | 1,267,922 | 1,312,303 |
| Deaths unreported by CR | 32,150 | 38,322 |
| Completeness – CR (%) | 97.5 | 97.1 |
| Deaths unreported by SIM | 3,747 | 2,531 |
| Completeness – SIM (%) | 99.7 | 99.8 |
| Deaths unreported by both sources | -25,282* | -18,166* |
| **Empirical Method - Model 1 - CR** |  |  |
| Total deaths | 1,313,183 | 1,341,027 |
| Deaths unreported by CR | 77,411 | 67,046 |
| Completeness – CR (%) | 94.1 | 95.0 |
| **Empirical Method - Model 1 - SIM** |  |  |
| Total deaths | 1,321,922 | 1,359,304 |
| Deaths unreported by SIM | 57,747 | 49,532 |
| Completeness – SIM (%) | 95.6 | 96.4 |
| **Empirical Method - Model 2 - CR** |  |  |
| Total deaths | 1,309,333 | 1,346,382 |
| Deaths unreported by CR | 73,561 | 72,401 |
| Completeness – CR (%) | 94.4 | 94.6 |
| **Empirical Method - Model 2 - SIM** |  |  |
| Total deaths - SIM | 1,331,042 | 1,374,660 |
| Deaths unreported by SIM | 66,867 | 64,888 |
| Completeness – SIM (%) | 95.0 | 95.3 |

* GBD estimated total deaths are less than the total deaths reported by the two data sources. Total deaths according to the empirical method are reported deaths (either CR or SIM) divided by estimated completeness.

**Supplementary Table 3: Results of GLM models of CR death reporting, 2015 and 2016**

|  | **2015** | | | **2016** | | |
| --- | --- | --- | --- | --- | --- | --- |
| **Parameter** | **Estimate** | **Standard Error** | **Pr > ChiSq** | **Estimate** | **Standard Error** | **Pr > ChiSq** |
| Intercept | 2.42 | 0.08 | <.0001 | 2.74 | 0.09 | <.0001 |
| **Sex** |  |  |  |  |  |  |
| Male | Ref. |  |  | Ref. |  |  |
| Female | -0.11 | 0.01 | <.0001 | -0.12 | 0.01 | <.0001 |
| Ignored | -1.00 | 0.10 | <.0001 | -0.47 | 0.11 | <.0001 |
| **Cause of death** |  |  |  |  |  |  |
| Natural | Ref. |  |  | Ref. |  |  |
| Unnatural | 0.12 | 0.02 | <.0001 | 0.08 | 0.02 | <.0001 |
| Ignored | 20.27 | 2032.92 | 0.99 | 20.17 | 2139.06 | 0.99 |
| **Age at death** |  |  |  |  |  |  |
| Neonatal | Ref. |  |  | Ref. |  |  |
| Post neonatal | 0.37 | 0.04 | <.0001 | 0.33 | 0.04 | <.0001 |
| 1-4 | 0.60 | 0.05 | <.0001 | 0.62 | 0.05 | <.0001 |
| 5-9 | 0.92 | 0.07 | <.0001 | 1.01 | 0.07 | <.0001 |
| 10-14 | 1.06 | 0.06 | <.0001 | 0.97 | 0.06 | <.0001 |
| 15-19 | 1.01 | 0.04 | <.0001 | 1.03 | 0.04 | <.0001 |
| 20-24 | 1.16 | 0.03 | <.0001 | 1.15 | 0.03 | <.0001 |
| 25-29 | 1.27 | 0.03 | <.0001 | 1.27 | 0.04 | <.0001 |
| 30-34 | 1.35 | 0.03 | <.0001 | 1.32 | 0.03 | <.0001 |
| 35-39 | 1.36 | 0.03 | <.0001 | 1.40 | 0.03 | <.0001 |
| 40-44 | 1.35 | 0.03 | <.0001 | 1.38 | 0.03 | <.0001 |
| 45-49 | 1.42 | 0.03 | <.0001 | 1.42 | 0.03 | <.0001 |
| 50-54 | 1.45 | 0.03 | <.0001 | 1.47 | 0.03 | <.0001 |
| 55-59 | 1.52 | 0.03 | <.0001 | 1.47 | 0.03 | <.0001 |
| 60-64 | 1.55 | 0.03 | <.0001 | 1.52 | 0.03 | <.0001 |
| 65-69 | 1.48 | 0.03 | <.0001 | 1.48 | 0.03 | <.0001 |
| 70-74 | 1.44 | 0.02 | <.0001 | 1.42 | 0.03 | <.0001 |
| 75-79 | 1.36 | 0.02 | <.0001 | 1.33 | 0.02 | <.0001 |
| 80-84 | 1.34 | 0.02 | <.0001 | 1.28 | 0.02 | <.0001 |
| 85+ | 1.27 | 0.02 | <.0001 | 1.20 | 0.02 | <.0001 |
| Ignored | -0.55 | 0.06 | <.0001 | -0.92 | 0.07 | <.0001 |

**Supplementary Table 3 (contd.)**

|  | **2015** | | | **2016** | | |
| --- | --- | --- | --- | --- | --- | --- |
| **Parameter** | **Estimate** | **Standard Error** | **Pr > ChiSq** | **Estimate** | **Standard Error** | **Pr > ChiSq** |
| **Place of death** |  |  |  |  |  |  |
| Hospital | Ref. |  |  | Ref. |  |  |
| Other health facilities | -0.15 | 0.03 | <.0001 | -0.06 | 0.03 | 0.02 |
| Household | -0.19 | 0.01 | <.0001 | -0.17 | 0.01 | <.0001 |
| Public place | -0.19 | 0.02 | <.0001 | -0.21 | 0.02 | <.0001 |
| Others | -0.52 | 0.02 | <.0001 | -0.54 | 0.02 | <.0001 |
| Ignored | -0.69 | 0.08 | <.0001 | -0.86 | 0.08 | <.0001 |
| **Municipality education decile** |  |  |  |  |  |  |
| 1 | Ref. |  |  | Ref. |  |  |
| 2 | 0.10 | 0.01 | <.0001 | 0.12 | 0.01 | <.0001 |
| 3 | 0.22 | 0.02 | <.0001 | 0.22 | 0.02 | <.0001 |
| 4 | 0.25 | 0.02 | <.0001 | 0.30 | 0.02 | <.0001 |
| 5 | 0.59 | 0.02 | <.0001 | 0.65 | 0.02 | <.0001 |
| 6 | 0.52 | 0.02 | <.0001 | 0.54 | 0.03 | <.0001 |
| 7 | 1.01 | 0.03 | <.0001 | 1.07 | 0.03 | <.0001 |
| 8 | 1.21 | 0.04 | <.0001 | 1.23 | 0.04 | <.0001 |
| 9 | 0.96 | 0.03 | <.0001 | 0.60 | 0.03 | <.0001 |
| 10 | 0.61 | 0.03 | <.0001 | 0.85 | 0.04 | <.0001 |
| Ignored | 0.27 | 0.07 | 0.00 | 0.29 | 0.07 | <.0001 |
| **Population density decile** |  |  |  |  |  |  |
| 1 | Ref. |  |  | Ref. |  |  |
| 2 | 0.09 | 0.02 | <.0001 | 0.06 | 0.02 | 0.00 |
| 3 | 0.08 | 0.02 | <.0001 | 0.06 | 0.02 | 0.00 |
| 4 | 0.06 | 0.02 | 0.00 | 0.05 | 0.02 | 0.00 |
| 5 | 0.27 | 0.02 | <.0001 | 0.18 | 0.02 | <.0001 |
| 6 | 0.18 | 0.03 | <.0001 | 0.14 | 0.03 | <.0001 |
| 7 | 0.48 | 0.03 | <.0001 | 0.35 | 0.03 | <.0001 |
| 8 | 0.10 | 0.03 | 0.00 | 0.24 | 0.03 | <.0001 |
| 9 | -0.33 | 0.04 | <.0001 | -0.13 | 0.04 | 0.00 |
| 10 | 0.57 | 0.04 | <.0001 | 0.55 | 0.05 | <.0001 |
| Ignored | 0.00 | 0.00 | . | 0.00 | 0.00 | . |

**Supplementary Table 3 (contd.)**

|  | **2015** | | | **2016** | | |
| --- | --- | --- | --- | --- | --- | --- |
| **Parameter** | **Estimate** | **Standard Error** | **Parameter** | **Estimate** | **Standard Error** | **Parameter** |
| **State of residence** |  |  |  |  |  |  |
| Rondônia | Ref. |  |  | Ref. |  |  |
| Acre | -0.94 | 0.11 | <.0001 | -1.05 | 0.12 | <.0001 |
| Amazonas | -2.10 | 0.08 | <.0001 | -2.37 | 0.09 | <.0001 |
| Roraima | -1.75 | 0.11 | <.0001 | -2.24 | 0.11 | <.0001 |
| Pará | -2.42 | 0.08 | <.0001 | -2.75 | 0.09 | <.0001 |
| Amapá | -2.81 | 0.09 | <.0001 | -3.16 | 0.10 | <.0001 |
| Tocantins | -1.81 | 0.09 | <.0001 | -2.03 | 0.10 | <.0001 |
| Maranhão | -2.94 | 0.08 | <.0001 | -3.25 | 0.09 | <.0001 |
| Piauí | -1.91 | 0.08 | <.0001 | -2.15 | 0.09 | <.0001 |
| Ceará | -1.58 | 0.08 | <.0001 | -1.81 | 0.09 | <.0001 |
| Rio Grande do Norte | -1.98 | 0.08 | <.0001 | -2.21 | 0.09 | <.0001 |
| Paraíba | -1.33 | 0.08 | <.0001 | -1.06 | 0.09 | <.0001 |
| Pernambuco | -0.85 | 0.08 | <.0001 | -1.20 | 0.09 | <.0001 |
| Alagoas | -1.51 | 0.08 | <.0001 | -1.81 | 0.09 | <.0001 |
| Sergipe | -1.55 | 0.08 | <.0001 | -1.77 | 0.09 | <.0001 |
| Bahia | -1.47 | 0.08 | <.0001 | -1.76 | 0.09 | <.0001 |
| Minas Gerais | -0.38 | 0.08 | <.0001 | -0.39 | 0.09 | <.0001 |
| Espírito Santo | -0.61 | 0.09 | <.0001 | 0.38 | 0.12 | 0.00 |
| Rio de Janeiro | -0.10 | 0.08 | 0.22 | -0.38 | 0.09 | <.0001 |
| São Paulo | 0.69 | 0.08 | <.0001 | 0.29 | 0.09 | 0.00 |
| Paraná | 0.07 | 0.08 | 0.37 | -0.02 | 0.09 | 0.86 |
| Santa Catarina | -0.69 | 0.08 | <.0001 | -0.93 | 0.09 | <.0001 |
| Rio Grande do Sul | 0.80 | 0.09 | <.0001 | 0.48 | 0.10 | <.0001 |
| Mato Grosso do Sul | -0.62 | 0.09 | <.0001 | -0.82 | 0.10 | <.0001 |
| Mato Grosso | -1.24 | 0.08 | <.0001 | -2.03 | 0.09 | <.0001 |
| Goiás | -0.95 | 0.08 | <.0001 | -1.12 | 0.09 | <.0001 |
| Distrito Federal | -0.12 | 0.11 | 0.28 | 0.42 | 0.16 | 0.01 |
| Ignored | 19.17 | 3154.94 | 1.00 | 19.15 | 3103.88 | 1.00 |

Ref.: Reference category

**Supplementary Table 4: Results of GLM models of SIM death reporting, 2015 and 2016**

|  | **2015** | | | **2016** | | |
| --- | --- | --- | --- | --- | --- | --- |
| **Parameter** | **Estimate** | **Standard Error** | **Pr > ChiSq** | **Estimate** | **Standard Error** | **Pr > ChiSq** |
| Intercept | 3.11 | 0.07 | <.0001 | 3.61 | 0.09 | <.0001 |
| **Sex** |  |  |  |  |  |  |
| Male | Ref. |  |  | Ref. |  |  |
| Female | 0.02 | 0.01 | 0.13 | -0.01 | 0.02 | 0.69 |
| Ignored | 0.19 | 0.22 | 0.39 | 0.34 | 0.33 | 0.31 |
| **Cause of death** |  |  |  |  |  |  |
| Natural | Ref. |  |  | Ref. |  |  |
| Unnatural | 0.61 | 0.03 | <.0001 | 0.67 | 0.03 | <.0001 |
| Ignored | -26.34 | 3142.47 | 0.99 | -26.61 | 3217.99 | 0.99 |
| **Age at death** |  |  |  |  |  |  |
| Neonatal | Ref. |  |  | Ref. |  |  |
| Post neonatal | 0.31 | 0.08 | 0.00 | 0.24 | 0.10 | 0.01 |
| 1-4 | -0.23 | 0.09 | 0.01 | 0.10 | 0.11 | 0.37 |
| 5-9 | -0.21 | 0.11 | 0.05 | 0.18 | 0.15 | 0.22 |
| 10-14 | -0.08 | 0.10 | 0.44 | 0.08 | 0.12 | 0.49 |
| 15-19 | 0.03 | 0.07 | 0.64 | 0.18 | 0.08 | 0.03 |
| 20-24 | -0.04 | 0.06 | 0.52 | 0.09 | 0.07 | 0.25 |
| 25-29 | -0.07 | 0.06 | 0.22 | 0.00 | 0.07 | 0.95 |
| 30-34 | -0.03 | 0.06 | 0.55 | 0.04 | 0.07 | 0.62 |
| 35-39 | 0.00 | 0.06 | 0.95 | 0.11 | 0.07 | 0.12 |
| 40-44 | 0.02 | 0.06 | 0.74 | 0.15 | 0.07 | 0.03 |
| 45-49 | 0.11 | 0.05 | 0.04 | 0.19 | 0.06 | 0.00 |
| 50-54 | 0.18 | 0.05 | 0.00 | 0.19 | 0.06 | 0.00 |
| 55-59 | 0.18 | 0.05 | 0.00 | 0.20 | 0.06 | 0.00 |
| 60-64 | 0.13 | 0.05 | 0.01 | 0.19 | 0.06 | 0.00 |
| 65-69 | 0.13 | 0.05 | 0.01 | 0.20 | 0.06 | 0.00 |
| 70-74 | 0.13 | 0.05 | 0.01 | 0.22 | 0.06 | <.0001 |
| 75-79 | 0.11 | 0.05 | 0.02 | 0.16 | 0.06 | 0.00 |
| 80-84 | 0.09 | 0.05 | 0.06 | 0.15 | 0.06 | 0.01 |
| 85+ | 0.02 | 0.05 | 0.62 | 0.08 | 0.05 | 0.14 |
| Ignored | -0.78 | 0.11 | <.0001 | -0.69 | 0.14 | <.0001 |

**Supplementary Table 4 (contd.)**

|  | **2015** | | | **2016** | | |
| --- | --- | --- | --- | --- | --- | --- |
| **Parameter** | **Estimate** | **Standard Error** | **Pr > ChiSq** | **Estimate** | **Standard Error** | **Pr > ChiSq** |
| **Place of death** |  |  |  |  |  |  |
| Hospital | Ref. |  |  | Ref. |  |  |
| Other health facilities | -0.45 | 0.03 | <.0001 | -0.34 | 0.04 | <.0001 |
| Household | -0.99 | 0.01 | <.0001 | -1.10 | 0.02 | <.0001 |
| Public place | -0.79 | 0.03 | <.0001 | -0.99 | 0.04 | <.0001 |
| Others | -0.83 | 0.03 | <.0001 | -0.67 | 0.04 | <.0001 |
| Ignored | -1.25 | 0.10 | <.0001 | -1.35 | 0.12 | <.0001 |
| **Municipality education decile** |  |  |  |  |  |  |
| 1 | Ref. |  |  | Ref. |  |  |
| 2 | -0.08 | 0.02 | <.0001 | -0.03 | 0.02 | 0.19 |
| 3 | 0.11 | 0.02 | <.0001 | 0.16 | 0.03 | <.0001 |
| 4 | 0.36 | 0.03 | <.0001 | 0.41 | 0.03 | <.0001 |
| 5 | 0.40 | 0.03 | <.0001 | 0.39 | 0.04 | <.0001 |
| 6 | 0.42 | 0.03 | <.0001 | 0.55 | 0.04 | <.0001 |
| 7 | 0.69 | 0.04 | <.0001 | 0.63 | 0.05 | <.0001 |
| 8 | 0.86 | 0.05 | <.0001 | 0.79 | 0.06 | <.0001 |
| 9 | 0.34 | 0.05 | <.0001 | 0.18 | 0.05 | 0.00 |
| 10 | -0.11 | 0.05 | 0.01 | 0.00 | 0.05 | 0.94 |
| Ignored | 0.22 | 0.11 | 0.06 | 0.60 | 0.16 | 0.00 |
| **Municipality population density decile** |  |  |  |  |  |  |
| 1 | Ref. |  |  | Ref. |  |  |
| 2 | 0.23 | 0.02 | <.0001 | 0.33 | 0.03 | <.0001 |
| 3 | 0.31 | 0.02 | <.0001 | 0.44 | 0.03 | <.0001 |
| 4 | 0.27 | 0.03 | <.0001 | 0.32 | 0.03 | <.0001 |
| 5 | 0.45 | 0.03 | <.0001 | 0.60 | 0.04 | <.0001 |
| 6 | 0.41 | 0.03 | <.0001 | 0.51 | 0.04 | <.0001 |
| 7 | 0.32 | 0.04 | <.0001 | 0.31 | 0.05 | <.0001 |
| 8 | 0.62 | 0.04 | <.0001 | 0.67 | 0.05 | <.0001 |
| 9 | -0.22 | 0.05 | <.0001 | -0.05 | 0.06 | 0.44 |
| 10 | 0.97 | 0.06 | <.0001 | 1.37 | 0.08 | <.0001 |
| Ignored | 0.00 | 0.00 | . | 0.00 | 0.00 | . |

**Supplementary Table 4 (contd.)**

|  | **2015** | | | **2016** | | |
| --- | --- | --- | --- | --- | --- | --- |
| **Parameter** | **Estimate** | **Standard Error** | **Pr > ChiSq** | **Estimate** | **Standard Error** | **Pr > ChiSq** |
| **State of residence** |  |  |  |  |  |  |
| Rondônia | Ref. |  |  | Ref. |  |  |
| Acre | -0.13 | 0.10 | 0.18 | -0.04 | 0.13 | 0.74 |
| Amazonas | -0.21 | 0.07 | 0.00 | -0.33 | 0.09 | 0.00 |
| Roraima | 0.31 | 0.15 | 0.03 | -0.70 | 0.13 | <.0001 |
| Pará | -0.20 | 0.06 | 0.00 | -0.44 | 0.08 | <.0001 |
| Amapá | -0.86 | 0.09 | <.0001 | -1.07 | 0.11 | <.0001 |
| Tocantins | -0.06 | 0.08 | 0.45 | -0.17 | 0.10 | 0.11 |
| Maranhão | -0.35 | 0.06 | <.0001 | -0.67 | 0.08 | <.0001 |
| Piauí | 0.49 | 0.07 | <.0001 | 0.65 | 0.10 | <.0001 |
| Ceará | -0.09 | 0.06 | 0.18 | -0.54 | 0.08 | <.0001 |
| Rio Grande do Norte | -0.27 | 0.07 | <.0001 | -0.63 | 0.09 | <.0001 |
| Paraíba | -0.47 | 0.07 | <.0001 | -0.57 | 0.09 | <.0001 |
| Pernambuco | 0.37 | 0.07 | <.0001 | 0.21 | 0.09 | 0.01 |
| Alagoas | -0.30 | 0.07 | <.0001 | -0.61 | 0.09 | <.0001 |
| Sergipe | 0.64 | 0.09 | <.0001 | 0.37 | 0.11 | 0.00 |
| Bahia | -0.10 | 0.06 | 0.11 | -0.45 | 0.08 | <.0001 |
| Minas Gerais | 0.27 | 0.06 | <.0001 | 0.16 | 0.08 | 0.04 |
| Espírito Santo | 0.57 | 0.08 | <.0001 | 0.41 | 0.10 | <.0001 |
| Rio de Janeiro | 1.66 | 0.07 | <.0001 | 1.37 | 0.09 | <.0001 |
| São Paulo | 2.93 | 0.08 | <.0001 | 2.46 | 0.10 | <.0001 |
| Paraná | 1.34 | 0.07 | <.0001 | 1.37 | 0.09 | <.0001 |
| Santa Catarina | 0.51 | 0.07 | <.0001 | 0.06 | 0.09 | 0.47 |
| Rio Grande do Sul | 1.13 | 0.07 | <.0001 | 0.87 | 0.09 | <.0001 |
| Mato Grosso do Sul | 0.40 | 0.08 | <.0001 | 0.18 | 0.10 | 0.07 |
| Mato Grosso | 0.26 | 0.07 | 0.00 | 0.17 | 0.09 | 0.08 |
| Goiás | 0.40 | 0.07 | <.0001 | 0.40 | 0.09 | <.0001 |
| Distrito Federal | 0.73 | 0.10 | <.0001 | 1.04 | 0.15 | <.0001 |
| Ignored | -25.85 | 4726.64 | 1.00 | -26.52 | 4515.44 | 1.00 |

Ref. = Reference category

**Supplementary Table 5: Completeness of death reporting (%) by source and method, Brazil and each state, 2015**

|  | **CR** | | | | **SIM** | | | | |
| --- | --- | --- | --- | --- | --- | --- | --- | --- | --- |
|  | **GLM** | **Empirical – model 1** | **Empirical – model 2** | **GBD** | **GLM** | **Empirical – model 1** | **Empirical – model 2** | **GBD** |  |
| Brazil | 95.4 | 94.1 | 94.4 | 97.5 | 97.6 | 95.6 | 95.0 | 99.7 |  |
| Rondônia | 97.8 | 97.0 | 94.5 | 96.9 | 95.9 | 96.9 | 94.4 | 95.0 |  |
| Acre | 94.1 | 88.5 | 86.2 | 96.4 | 95.2 | 89.2 | 86.7 | 97.5 |  |
| Amazonas | 88.8 | 91.2 | 90.4 | 93.6 | 95.5 | 95.6 | 92.1 | 100.7 |  |
| Roraima | 90.5 | 86.7 | 86.5 | 90.2 | 96.9 | 91.4 | 88.6 | 96.6 |  |
| Pará | 81.5 | 85.7 | 88.3 | 86.2 | 93.8 | 95.0 | 92.1 | 99.2 |  |
| Amapá | 79.9 | 83.9 | 84.5 | 89.8 | 90.5 | 92.3 | 88.2 | 101.7 |  |
| Tocantins | 89.8 | 88.5 | 89.7 | 90.6 | 95.2 | 92.6 | 91.3 | 96.1 |  |
| Maranhão | 71.3 | 70.9 | 78.7 | 71.6 | 91.7 | 89.7 | 88.2 | 92.1 |  |
| Piauí | 87.3 | 83.1 | 89.1 | 90.3 | 96.5 | 93.1 | 92.3 | 99.8 |  |
| Ceará | 92.2 | 88.8 | 92.2 | 96.3 | 95.6 | 92.2 | 93.4 | 99.9 |  |
| Rio Grande do Norte | 88.1 | 93.9 | 93.4 | 92.2 | 94.5 | 96.8 | 94.9 | 98.9 |  |
| Paraíba | 93.0 | 94.7 | 95.3 | 96.9 | 93.7 | 96.1 | 95.7 | 97.6 |  |
| Pernambuco | 96.0 | 92.9 | 94.6 | 97.6 | 97.4 | 95.0 | 95.4 | 99.1 |  |
| Alagoas | 91.3 | 94.0 | 94.8 | 94.2 | 94.5 | 96.6 | 95.5 | 97.5 |  |
| Sergipe | 91.8 | 92.2 | 93.3 | 95.2 | 97.8 | 95.6 | 94.7 | 101.4 |  |
| Bahia | 91.7 | 89.8 | 91.4 | 92.8 | 94.6 | 93.6 | 92.4 | 95.7 |  |
| Minas Gerais | 97.6 | 94.8 | 94.4 | 100.6 | 97.4 | 95.2 | 94.7 | 100.4 |  |
| Espírito Santo | 97.2 | 95.5 | 94.2 | 99.4 | 98.2 | 95.4 | 94.6 | 100.4 |  |
| Rio de Janeiro | 98.4 | 97.4 | 96.8 | 99.8 | 99.3 | 97.6 | 97.2 | 100.7 |  |
| São Paulo | 99.5 | 97.0 | 96.3 | 101.1 | 99.9 | 97.1 | 96.5 | 101.5 |  |
| Paraná | 98.5 | 96.6 | 96.0 | 99.2 | 99.0 | 96.8 | 96.2 | 99.6 |  |
| Santa Catarina | 97.4 | 91.7 | 92.8 | 98.5 | 98.1 | 92.6 | 93.3 | 99.3 |  |
| Rio Grande do Sul | 99.4 | 97.7 | 97.0 | 100.2 | 99.0 | 97.8 | 97.2 | 99.8 |  |
| Mato Grosso do Sul | 97.0 | 97.9 | 96.3 | 99.2 | 97.7 | 98.2 | 96.6 | 99.9 |  |
| Mato Grosso | 94.0 | 97.1 | 95.0 | 97.2 | 96.9 | 98.0 | 95.5 | 100.2 |  |
| Goiás | 95.8 | 94.3 | 94.6 | 99.7 | 97.8 | 95.6 | 95.2 | 101.7 |  |
| Distrito Federal | 98.6 | 94.4 | 92.2 | 100.9 | 98.2 | 94.4 | 92.5 | 100.6 |  |
| Root mean squared difference with GLM | - | 2.7 | 3.0 | 2.6 | - | 2.4 | 3.0 | 2.8 |  |

**Supplementary Table 6: Completeness of death reporting (%) by source using GLM, by various population-sub-groups, 2015 and 2016**

|  | **CR** | | **SIM** | |
| --- | --- | --- | --- | --- |
|  | **2015** | **2016** | **2015** | **2016** |
| **Sex** |  |  |  |  |
| Male | 95.3 | 95.6 | 98.3 | 97.5 |
| Female | 95.4 | 95.7 | 98.4 | 97.7 |
| Ignored | 68.9 | 75.1 | 91.2 | 89.6 |
| **Place of death** |  |  |  |  |
| Hospital | 96.2 | 96.4 | 99.0 | 98.5 |
| Other health facility | 97.0 | 97.3 | 98.9 | 98.3 |
| Household | 93.3 | 93.9 | 96.0 | 94.6 |
| Public place | 93.4 | 93.4 | 97.5 | 96.8 |
| Other facility | 92.2 | 92.1 | 97.9 | 96.3 |
| Ignored | 84.1 | 81.1 | 88.1 | 85.5 |
| **Cause of death** |  |  |  |  |
| Natural | 95.5 | 95.8 | 98.4 | 97.6 |
| Unnatural | 94.3 | 94.2 | 98.5 | 97.8 |
| Ignored | 100.0 | 100.0 | - | - |
| **Age group** |  |  |  |  |
| Neonatal | 83.7 | 84.3 | 97.9 | 97.2 |
| Post neonatal | 87.3 | 87.4 | 98.2 | 97.7 |
| 01-04 | 87.7 | 88.6 | 97.8 | 95.9 |
| 05-09 | 90.1 | 91.1 | 97.9 | 96.1 |
| 10-14 | 91.9 | 91.4 | 98.0 | 96.8 |
| 15-19 | 92.7 | 92.7 | 98.3 | 97.4 |
| 20-24 | 93.6 | 93.4 | 98.2 | 97.4 |
| 25-29 | 93.9 | 94.1 | 98.0 | 97.1 |
| 30-34 | 94.5 | 94.6 | 98.0 | 97.2 |
| 35-39 | 94.9 | 95.3 | 98.2 | 97.3 |
| 40-44 | 95.4 | 95.7 | 98.3 | 97.4 |
| 45-49 | 95.9 | 96.1 | 98.4 | 97.7 |
| 50-54 | 96.3 | 96.6 | 98.5 | 98.0 |
| 55-59 | 96.7 | 96.8 | 98.6 | 98.1 |
| 60-64 | 96.8 | 97.0 | 98.6 | 97.9 |
| 65-69 | 96.5 | 96.8 | 98.6 | 97.9 |
| 70-74 | 96.3 | 96.5 | 98.5 | 97.8 |
| 75-79 | 95.9 | 96.2 | 98.4 | 97.7 |
| 80-84 | 95.9 | 96.1 | 98.3 | 97.6 |
| 85+ | 95.3 | 95.6 | 97.9 | 97.0 |
| Ignored | 77.8 | 74.7 | 91.3 | 90.5 |

**Supplementary Table 6 (contd.)**

|  | **CR** | | **SIM** | |
| --- | --- | --- | --- | --- |
|  | **2015** | **2016** | **2015** | **2016** |
| **State of residence** |  |  |  |  |
| Rondônia | 97.8 | 98.4 | 97.7 | 95.9 |
| Acre | 94.1 | 95.4 | 97.5 | 95.2 |
| Amazonas | 88.8 | 89.1 | 96.9 | 95.5 |
| Roraima | 90.5 | 86.7 | 95.0 | 96.9 |
| Pará | 81.5 | 81.3 | 95.6 | 93.8 |
| Amapá | 79.9 | 75.9 | 92.6 | 90.5 |
| Tocantins | 89.8 | 90.8 | 97.0 | 95.2 |
| Maranhão | 71.3 | 71.5 | 93.7 | 91.7 |
| Piauí | 87.3 | 87.8 | 98.3 | 96.5 |
| Ceará | 92.2 | 92.6 | 96.4 | 95.6 |
| Rio Grande do Norte | 88.1 | 89.1 | 95.8 | 94.5 |
| Paraíba | 93.0 | 96.0 | 96.2 | 93.7 |
| Pernambuco | 96.0 | 95.8 | 98.4 | 97.4 |
| Alagoas | 91.3 | 91.6 | 95.8 | 94.5 |
| Sergipe | 91.8 | 92.1 | 98.5 | 97.8 |
| Bahia | 91.7 | 91.6 | 95.8 | 94.6 |
| Minas Gerais | 97.6 | 98.3 | 98.5 | 97.4 |
| Espírito Santo | 97.2 | 99.3 | 98.9 | 98.2 |
| Rio de Janeiro | 98.4 | 98.3 | 99.5 | 99.3 |
| São Paulo | 99.5 | 99.5 | 99.9 | 99.9 |
| Paraná | 98.5 | 98.8 | 99.5 | 99.0 |
| Santa Catarina | 97.4 | 97.5 | 98.4 | 98.1 |
| Rio Grande do Sul | 99.4 | 99.4 | 99.3 | 99.0 |
| Mato Grosso do Sul | 97.0 | 97.3 | 98.3 | 97.7 |
| Mato Grosso | 94.0 | 90.5 | 98.0 | 96.9 |
| Goiás | 95.8 | 96.2 | 98.8 | 97.8 |
| Distrito Federal | 98.6 | 99.5 | 99.4 | 98.2 |

**Supplementary Table 6 (contd.)**

|  | **CR** | | **SIM** | |
| --- | --- | --- | --- | --- |
|  | **2015** | **2016** | **2015** | **2016** |
| **Municipality education level decile** |  |  |  |  |
| 1 | 86.9 | 87.3 | 95.5 | 93.8 |
| 2 | 90.4 | 91.0 | 96.1 | 94.5 |
| 3 | 93.7 | 94.1 | 97.8 | 96.7 |
| 4 | 95.6 | 96.0 | 98.8 | 98.2 |
| 5 | 97.5 | 97.8 | 99.0 | 98.6 |
| 6 | 97.1 | 97.3 | 99.2 | 98.7 |
| 7 | 98.3 | 98.5 | 99.4 | 99.1 |
| 8 | 98.8 | 98.8 | 99.6 | 99.3 |
| 9 | 97.6 | 97.1 | 98.8 | 98.5 |
| 10 | 98.0 | 98.5 | 99.2 | 98.6 |
| Ignored | 88.3 | 86.7 | 88.2 | 86.2 |
| **Muncipality population density decile** |  |  |  |  |
| 1 | 89.4 | 89.7 | 96.0 | 94.4 |
| 2 | 91.7 | 92.1 | 97.2 | 96.0 |
| 3 | 93.2 | 93.7 | 97.8 | 96.7 |
| 4 | 94.4 | 94.8 | 97.8 | 97.0 |
| 5 | 96.2 | 96.1 | 98.7 | 98.0 |
| 6 | 97.5 | 97.7 | 99.1 | 98.6 |
| 7 | 97.2 | 97.3 | 98.9 | 98.5 |
| 8 | 97.7 | 98.2 | 99.3 | 99.0 |
| 9 | 97.4 | 97.5 | 98.7 | 98.2 |
| 10 | 99.3 | 99.4 | 99.8 | 99.6 |
| Ignored | 88.3 | 86.7 | 88.2 | 86.2 |

**Supplementary Table 7: Estimated deaths not captured by either system, captured by CR and captured by SIM using GLM (%), by various population sub-groups, 2015 and 2016**

|  | **2015** | | | **2016** | | |
| --- | --- | --- | --- | --- | --- | --- |
|  | **Deaths in neither system** | **CR deaths** | **SIM deaths** | **Deaths in neither system** | **CR deaths** | **SIM deaths** |
| **Sex** |  |  |  |  |  |  |
| Male | 57.1 | 56.1 | 56.1 | 57.1 | 56.2 | 56.3 |
| Female | 42.4 | 43.8 | 43.9 | 42.6 | 43.7 | 43.7 |
| Ignored | 0.5 | 0.0 | 0.1 | 0.2 | 0.0 | 0.0 |
| **Place of death** |  |  |  |  |  |  |
| Hospital | 33.1 | 67.0 | 67.1 | 32.2 | 67.1 | 67.1 |
| Other health facilities | 2.5 | 5.0 | 4.9 | 2.3 | 5.3 | 5.3 |
| Household | 49.4 | 19.9 | 19.7 | 51.0 | 19.7 | 19.6 |
| Public place | 7.1 | 4.8 | 4.8 | 7.6 | 4.5 | 4.6 |
| Others | 6.8 | 3.2 | 3.3 | 5.6 | 3.3 | 3.4 |
| Ignored | 1.1 | 0.1 | 0.1 | 1.3 | 0.1 | 0.1 |
| **Cause of death** |  |  |  |  |  |  |
| Natural | 88.1 | 88.0 | 88.0 | 88.2 | 88.2 | 88.1 |
| Unnatural | 11.9 | 11.9 | 12.0 | 11.8 | 11.7 | 11.9 |
| Ignored | 0.0 | 0.1 | 0.0 | 0.0 | 0.1 | 0.0 |
| **Age group** |  |  |  |  |  |  |
| Neonatal | 6.0 | 1.8 | 2.1 | 5.9 | 1.7 | 1.9 |
| Post neonatal | 1.8 | 0.8 | 0.9 | 2.0 | 0.8 | 0.9 |
| 1-4 | 1.6 | 0.4 | 0.4 | 1.3 | 0.4 | 0.5 |
| 5-9 | 0.7 | 0.2 | 0.3 | 0.5 | 0.2 | 0.3 |
| 10-14 | 0.7 | 0.4 | 0.4 | 0.7 | 0.4 | 0.4 |
| 15-19 | 2.2 | 1.6 | 1.7 | 2.1 | 1.6 | 1.7 |
| 20-24 | 2.7 | 2.1 | 2.2 | 2.7 | 2.1 | 2.2 |
| 25-29 | 2.8 | 2.1 | 2.1 | 2.7 | 2.1 | 2.1 |
| 30-34 | 2.8 | 2.4 | 2.4 | 2.8 | 2.3 | 2.3 |
| 35-39 | 2.9 | 2.7 | 2.7 | 2.7 | 2.6 | 2.7 |
| 40-44 | 3.0 | 3.1 | 3.1 | 2.8 | 3.1 | 3.1 |
| 45-49 | 3.2 | 4.1 | 4.1 | 3.2 | 4.0 | 4.0 |
| 50-54 | 3.6 | 5.4 | 5.4 | 3.7 | 5.4 | 5.3 |
| 55-59 | 3.9 | 6.7 | 6.6 | 4.2 | 6.8 | 6.7 |
| 60-64 | 4.9 | 7.9 | 7.8 | 4.8 | 8.0 | 8.0 |
| 65-69 | 6.0 | 8.9 | 8.8 | 6.1 | 9.1 | 9.0 |
| 70-74 | 7.3 | 9.5 | 9.5 | 7.1 | 9.6 | 9.6 |
| 75-79 | 9.5 | 10.8 | 10.8 | 9.4 | 10.7 | 10.7 |
| 80-84 | 9.7 | 10.6 | 10.6 | 10.1 | 10.7 | 10.7 |
| 85+ | 22.4 | 18.0 | 17.9 | 23.1 | 18.1 | 18.0 |
| Ignored | 2.3 | 0.2 | 0.3 | 2.0 | 0.2 | 0.2 |

**Supplementary Table 7 (contd.)**

|  | **2015** | | | **2016** | | |
| --- | --- | --- | --- | --- | --- | --- |
|  | **Deaths in neither system** | **CR deaths** | **SIM deaths** | **Deaths in neither system** | **CR deaths** | **SIM deaths** |
| **State of residence** |  |  |  |  |  |  |
| Rondônia | 0.3 | 0.7 | 0.6 | 0.2 | 0.7 | 0.6 |
| Acre | 0.4 | 0.3 | 0.3 | 0.2 | 0.3 | 0.3 |
| Amazonas | 4.1 | 1.3 | 1.3 | 3.7 | 1.2 | 1.3 |
| Roraima | 0.3 | 0.2 | 0.2 | 0.8 | 0.2 | 0.2 |
| Pará | 15.3 | 2.6 | 3.0 | 15.8 | 2.6 | 2.9 |
| Amapá | 1.9 | 0.2 | 0.2 | 2.2 | 0.2 | 0.2 |
| Tocantins | 1.4 | 0.6 | 0.6 | 1.1 | 0.6 | 0.6 |
| Maranhão | 25.7 | 2.1 | 2.7 | 27.5 | 2.1 | 2.6 |
| Piauí | 3.1 | 1.4 | 1.5 | 2.0 | 1.3 | 1.5 |
| Ceará | 7.7 | 4.3 | 4.4 | 8.5 | 4.1 | 4.1 |
| Rio Grande do Norte | 4.8 | 1.5 | 1.6 | 5.2 | 1.6 | 1.7 |
| Paraíba | 4.7 | 2.1 | 2.1 | 2.4 | 2.2 | 2.1 |
| Pernambuco | 2.6 | 5.0 | 4.9 | 2.5 | 5.1 | 5.1 |
| Alagoas | 3.4 | 1.5 | 1.6 | 3.7 | 1.6 | 1.6 |
| Sergipe | 0.9 | 1.0 | 1.1 | 0.8 | 1.0 | 1.0 |
| Bahia | 14.2 | 6.8 | 6.9 | 15.8 | 6.6 | 6.7 |
| Minas Gerais | 3.4 | 10.6 | 10.4 | 2.1 | 10.6 | 10.3 |
| Espírito Santo | 0.4 | 1.8 | 1.8 | 0.1 | 1.8 | 1.7 |
| Rio de Janeiro | 0.6 | 10.6 | 10.5 | 0.7 | 10.9 | 10.8 |
| São Paulo | 0.1 | 23.2 | 22.8 | 0.1 | 23.2 | 22.6 |
| Paraná | 0.5 | 5.7 | 5.6 | 0.3 | 5.8 | 5.7 |
| Santa Catarina | 0.8 | 3.1 | 3.0 | 0.9 | 3.1 | 3.1 |
| Rio Grande do Sul | 0.2 | 6.7 | 6.5 | 0.2 | 6.9 | 6.7 |
| Mato Grosso do Sul | 0.5 | 1.2 | 1.2 | 0.5 | 1.3 | 1.3 |
| Mato Grosso | 1.3 | 1.3 | 1.4 | 1.7 | 1.3 | 1.3 |
| Goiás | 1.5 | 3.1 | 3.1 | 1.0 | 2.9 | 2.9 |
| Distrito Federal | 0.1 | 1.0 | 0.9 | 0.0 | 0.9 | 0.9 |
| Ignored | 0.3 | 0.0 | 0.0 | 0.2 | 0.0 | 0.0 |

**Supplementary Table 7 (contd**.)

|  | **2015** | | | **2016** | | |
| --- | --- | --- | --- | --- | --- | --- |
|  | **Deaths in neither system** | **CR deaths** | **SIM deaths** | **Deaths in neither system** | **CR deaths** | **SIM deaths** |
| **Municipality education level decile** |  |  |  |  |  |  |
| 1 | 38.2 | 9.1 | 9.6 | 39.8 | 9.1 | 9.6 |
| 2 | 27.6 | 9.5 | 9.7 | 26.9 | 9.5 | 9.7 |
| 3 | 12.9 | 9.8 | 9.9 | 12.5 | 9.9 | 10.0 |
| 4 | 5.5 | 9.7 | 9.7 | 5.1 | 9.8 | 9.8 |
| 5 | 2.8 | 10.6 | 10.5 | 2.7 | 10.8 | 10.6 |
| 6 | 3.0 | 10.0 | 9.9 | 2.5 | 10.0 | 9.9 |
| 7 | 1.2 | 9.7 | 9.6 | 1.2 | 9.6 | 9.4 |
| 8 | 1.2 | 10.7 | 10.6 | 1.1 | 10.8 | 10.6 |
| 9 | 3.4 | 10.4 | 10.2 | 4.8 | 10.2 | 10.1 |
| 10 | 3.2 | 10.0 | 9.9 | 2.6 | 10.1 | 9.9 |
| Ignored | 1.1 | 0.3 | 0.3 | 0.8 | 0.3 | 0.3 |
| **Muncipality population density decile** |  |  |  |  |  |  |
| 1 | 32.2 | 9.4 | 9.7 | 33.4 | 9.3 | 9.6 |
| 2 | 21.2 | 9.6 | 9.8 | 21.3 | 9.6 | 9.8 |
| 3 | 15.2 | 9.8 | 10.0 | 14.4 | 9.8 | 10.0 |
| 4 | 12.0 | 9.9 | 9.9 | 12.6 | 9.9 | 9.9 |
| 5 | 5.9 | 10.1 | 10.0 | 5.4 | 10.1 | 10.1 |
| 6 | 3.0 | 10.9 | 10.8 | 2.6 | 11.0 | 10.8 |
| 7 | 4.3 | 9.8 | 9.7 | 4.3 | 9.8 | 9.7 |
| 8 | 1.8 | 10.2 | 10.1 | 1.4 | 10.3 | 10.1 |
| 9 | 3.2 | 9.8 | 9.6 | 3.6 | 9.9 | 9.7 |
| 10 | 0.3 | 10.2 | 10.0 | 0.2 | 10.1 | 9.9 |
| Ignored | 1.1 | 0.3 | 0.3 | 0.8 | 0.3 | 0.3 |

Note: Each variable in each column sums to 100%.

**Supplementary Table 8: Key mortality indicators calculated using GLM estimated deaths, by sex and state, Brazil, 2016**

|  | **Life expectancy at birth** | | | **Adult mortality (45q15), per 1,000** | | |
| --- | --- | --- | --- | --- | --- | --- |
| **State of residence** | **Male** | **Female** | **Both** | **Male** | **Female** | **Both** |
| Rondônia | 71.6 | 78.4 | 74.7 | 180 | 85 | 134 |
| Acre | 71.0 | 78.7 | 74.6 | 198 | 87 | 144 |
| Amazonas | 71.5 | 78.7 | 74.9 | 182 | 91 | 138 |
| Roraima | 71.0 | 77.6 | 74.1 | 181 | 90 | 137 |
| Pará | 71.2 | 79.2 | 74.9 | 193 | 90 | 143 |
| Amapá | 71.4 | 80.4 | 75.5 | 193 | 88 | 142 |
| Tocantins | 72.9 | 79.8 | 76.2 | 192 | 88 | 142 |
| Maranhão | 71.9 | 80.3 | 75.9 | 197 | 98 | 147 |
| Piauí | 71.7 | 79.6 | 75.6 | 191 | 85 | 138 |
| Ceará | 71.9 | 79.8 | 75.8 | 197 | 82 | 139 |
| Rio Grande do Norte | 70.8 | 79.6 | 75.1 | 208 | 85 | 147 |
| Paraíba | 71.0 | 78.1 | 74.6 | 202 | 93 | 146 |
| Pernambuco | 69.2 | 77.4 | 73.4 | 229 | 102 | 164 |
| Alagoas | 68.8 | 76.8 | 72.8 | 234 | 111 | 172 |
| Sergipe | 69.2 | 78.7 | 73.9 | 231 | 92 | 161 |
| Bahia | 71.0 | 80.0 | 75.4 | 212 | 94 | 153 |
| Minas Gerais | 73.4 | 80.3 | 76.8 | 181 | 89 | 135 |
| Espírito Santo | 73.1 | 81.1 | 77.0 | 184 | 84 | 135 |
| Rio de Janeiro | 70.2 | 77.9 | 74.1 | 206 | 109 | 157 |
| São Paulo | 73.2 | 80.0 | 76.6 | 170 | 85 | 127 |
| Paraná | 72.3 | 79.4 | 75.8 | 183 | 89 | 136 |
| Santa Catarina | 74.0 | 80.5 | 77.2 | 155 | 81 | 118 |
| Rio Grande do Sul | 72.3 | 79.7 | 76.0 | 183 | 91 | 137 |
| Mato Grosso do Sul | 71.6 | 78.5 | 74.9 | 193 | 95 | 144 |
| Mato Grosso | 71.6 | 79.1 | 75.1 | 195 | 89 | 144 |
| Goiás | 71.8 | 79.4 | 75.5 | 196 | 90 | 144 |
| Distrito Federal | 74.8 | 81.7 | 78.4 | 146 | 68 | 105 |
